# Sulfamethoxazole/Trimethoprim induced liver failure: a case report

**DOI:** 10.1186/1757-1626-1-44

**Published:** 2008-07-18

**Authors:** Salaheldin Abusin, Swapna Johnson

**Affiliations:** 1 Department of Medicine, Stroger Hospital of Cook County, 1901 W Harrison St, Chicago, IL, 60612, USA

## Abstract

Sulfamethoxazole/Trimethoprim (SMX/TMP) is a commonly used antibiotic, its' known adverse reaction of hepatotoxicity leading to acute liver failure is considered rare. We present a case of a 22 year old female who developed jaundice and acute liver failure one week after taking SMX/TMP for a UTI. After an extensive work up, a clinical diagnosis of SMX/TMP induced liver failure was reached. Over the course of several weeks she made a good clinical and biochemical recovery with supportive care. In this case report we describe her clinical presentation and course, and present a brief review of the literature.

## Introduction

Sulfamethoxazole/Trimethoprim (SMX/TMP) is a commonly used antibiotic for respiratory, gastrointestinal and urinary tract infections caused by a range of aerobic gram-positive and gram-negative bacteria. It also has activity against Listeria monocytogenes, Nocardia and Pneuomcystis jiroveci.

SMX/TMP is generally well tolerated in non-HIV-infected patients in whom adverse reactions occur in approximately 6 to 8 percent of individuals. In comparison, the adverse reaction rate is as high as 25 to 50 percent in HIV-infected patients, with many of the reactions being severe.

The most common adverse reactions include nausea, vomiting, anorexia, dermatological reactions such as pruritis, urticaria and less commonly Steven Johnson Syndrome. Life-threatening adverse reactions include neutropenia, exfoliative dermatitis (a severe skin disorder with generalized erythema and scaling) and toxic epidermal necrolysis (an acute severe reaction with widespread erythema and detachment of the epidermis). Acute liver failure has only been reported in a few cases worldwide, and has been attributed to the sulphonamide component of the drug.

## Case presentation

A previously healthy 22 year old female presented to our institution with nausea, vomiting and general malaise which started 2 days after taking SMX/TMP for a presumed urinary tract infection. Though feeling ill she continued her antibiotic course. On day 6 of SMX/TMP, her family noticed yellow discoloration of the sclera and urged her to go to the hospital. SMX/TMP was stopped on admission. On examination her only positive finding was generalized icterus most notably in the sclera, she had no signs of hepatic encephalopathy, or hepatomegaly. Her initial laboratory investigations showed an AST of 3077, ALT 4067, Alkaline Phosphatase 128, total bilirubin 5.1, INR 1.8, and albumin of 3.6. Over the course of her hospital stay her liver enzymes showed a downward trend, her total bilirubin peaked at 24.4, with direct bilirubin of 17.5, and INR at 2.16, after which they all gradually dropped. Ultrasound Liver with Doppler Flow showed normal liver size and echogenicity, with no obstruction of the biliary tree and normal blood flow. She was found to be negative for Hepatitis A, B, and C viruses, HIV, HSV and EBV. RPR, autoimmune panel, thyroid function, serum copper and ceruloplasmin were all within normal limits. Acetominophen was not detected in her blood. Based on the timing of jaundice in relation to SMX/TMP exposure, and the absence of any other identifiable cause, she was diagnosed with SMX/TMP induced acute liver failure. Her liver function improved with no intervention, and she made an uneventful recovery. At 2 months, she was asymptomatic, and her liver function tests including coagulation profile were back to normal.

## Discussion

The sulfamethoxazole component of SMX/TMP is responsible for most of its' side effects including liver failure. Although Trimethoprim alone can be used for treatment of uncomplicated UTI[1], SMX/TMP is commonly used for that purpose in the United States for unclear reasons.

Three forms of SMX/TMP induced liver damage have been described; hepatocellular[2], mixed hepatocellular cholestatic[3], and (more recently) bile duct injury with ductopenia or Vanishing Bile duct syndrome[4,5]. The onset of symptoms usually occurs within a few days of exposure as did in our patient, but can take up to a 1–2 months[6,7]. Patients usually present with jaundice, nausea, vomiting, and pruritis (if cholestatic). LFTs may show a hepatocellular or cholestatic pattern depending on the type of injury, coagulopathy is also seen. Patients might have other feature of an allergic reaction such as skin rash, eosinophilia[7,8]. Extrahepatic manifestations include pancytopenia[8], pancreatitis[9,10], and acute renal failure[7,8].

Diagnosis is suspected from the clinical presentation, and absence of other causes, in addition to suggestive changes on liver biopsy. Invitro tests such as lymphocyte transformation test have been reported to aid in the diagnosis[11]. Rechallenge with SMX/TMP as a method for diagnosis has been reported in the literature in the 1960s[12,13]. However, rechallenge even at small doses can lead to terminal hepatic failure[2], and it has fallen out of favor and only reported inadvertently since[14].

The severity of SMX/TMP induced liver injury can range from mild symptoms with elevated liver enzymes to fulminant hepatic failure with hepatic encephalopathy and coagulopathy. Outcome can be favorable with spontaneous resolution, as in our patient, or unfavorable leading to death reported in both HIV[15], (in this case it was associated with a delayed diagnosis) and non HIV infected patients[2,16].

Treatment is generally supportive, liver transplantation has been successful for both fulminant hepatic failure[17] and vanishing bile duct syndrome[4].

## Conclusion

This case illustrates a rare but clinically important side effect of a commonly used antibiotic. It alerts the physician to suspect drugs as a cause in any patient presenting with jaundice and/or acute liver failure. Failure to do so has been associated with a fatal outcome. It also shows that in the right clinical context, the diagnosis can be established without the need for an invasive liver biopsy in a patient with coagulopathy.

## Abbreviations

AST: Aspartate aminotransferase; ALT: Alanine aminotransferase; INR: International Normalized Ratio; HIV: Human Immunodeficiency virus; RPR: Rapid plasma reagin test for syphilis; HSV: Herpes Simplex Virus; EBV: Epstein Barr virus.

## Consent

Written informed consent was obtained from the patient for publication of this case report and accompanying images. A copy of the written consent is available for review by the Editor-in-Chief of this journal.

## Competing interests

The authors declare that they have no competing interests.

## Authors' contributions

SA and SJ were involved in the care of the patient during her hospital stay, and both analyzed and interpreted the patient data, and contributed to writing the manuscript. All authors read and approved the final manuscript.

## References

[B1] Hooton TM, Besser R, Foxman B, Fritsche TR, Nicolle LE Acute uncomplicated cystitis in an era of increasing antibiotic resistance: a proposed approach to empirical therapy. Clin Infect Dis.

[B2] Ransohoff DF, Jacobs G (1981). Terminal hepatic failure following a small dose of sulfamethoxazole-trimethoprim. Gastroenterology.

[B3] Abi Mansur P, Ardiaca MC, Allam C, Shammaia M (1981). Trimethoprim-sulfamethoxazole induced cholestasis. Am J Gastroenterol.

[B4] Yao F, Behling CA, Saab S, Li S, Hart M, Lyche KD (1997). trimethoprim-sulfamethoxazole induced vanishing bile duct syndrome. Am J Gastroenterology.

[B5] Altraif I, Lilly L, Wanless IR, Heathcote J (1994). Cholestatic liver disease with ductopenia (vanishing bile duct syndrome) after administration of clindamycin and trimethoprim-sulfamethoxazole. Am J Gastroenterol.

[B6] Mair SS, Kaplan JM, Levine LH, Geraci K (1981). Trimethoprim sulfamethoxazole induced cholestasis. Am J Gastroentertol.

[B7] Kraemer MJ, Kendall R, Hickman RO, Hass JE, Bierman CW (1982). a generalized allergic reaction with acute interstiial nephritis following trimethoprim-sulfamethoxazole use. Ann Allergy.

[B8] Windecker R, Steffen, Cascorbi I, Thurmann PA (2000). cotrimoxazole induced liver and renal failure. Eur J Clin Pharmacol.

[B9] Brett AS, Shaw SV (1999). Simultaneous pancreatitis and hepatitis associated with trimethoprim-sulfamethoxazole. Am J Gastroenterol.

[B10] Alberti-Flor JJ, Hernandez ME, Ferrer JP, Howell S, Jeffers L (1989). Fulminant liver failure and pancreatitis associated with the use of sulfamethoxazole-trimethoprim. Am J Gastroenterol.

[B11] Hofer T, Becker EW, Weigand K, Berg PA (1992). Demonstration of sensitized lymphocytes to trimethoprim/sulfamethoxazole and ofloxacin in a patient with cholestatic hepatitis. J Hepatol.

[B12] Fries J, Siraganian R (1966). Sulfonamide hepatitis. Report of a case due to sulfamethoxazole and sulfisoxazole. New Engl J Med.

[B13] Dujovne CA, Chan CH, Zimmerman HJ (1967). Sulfonamide hepatic injury. Review of the litertature and report of a case due to sulfamethoxazole. New Engl J Med.

[B14] Thies PW, Dull WL (1984). Trimethoprim-sulfamethoxazole induced cholestatic hepatitis. Arch Intern Med.

[B15] Kreisberg R Clinical problem-solving. We blew it. N Engl J Med.

[B16] Ilario MJ, Ruiz JE, Axiotis CA (2000). Acute fulminant hepatic failure in a woman treated with phenytoin and trimethoprim-sulfamethoxazole. Arch Pathol Lab Med.

[B17] Zaman F, Ye G, Abreo KD, Latif S, Zibari GB (2003). Successful orthotopic liver transplantation after trimethoprim-sulfamethoxazole associated fulminant liver failure. Clin Transplant.

